# Novel Intracoronary Infusion of Supersaturated Oxygen Therapy in Patients Presenting With Acute ST Elevation Myocardial Infarction: Does It Help?

**DOI:** 10.7759/cureus.39915

**Published:** 2023-06-03

**Authors:** Sardar M Alamzaib, Jay C Jensen, Kanaan Mansoor, Noor Ul Ann Rabbani, Rameez Sayyed

**Affiliations:** 1 Cardiology, Marshall University Joan C. Edwards School of Medicine, Huntington, USA; 2 Internal Medicine, Marshall University Joan C. Edwards School of Medicine, Huntington, USA

**Keywords:** echocardiogram (echo), myocardial infarction, early ptca (percutaneous transluminal coronary angioplasty), primary percutaneous coronary intervention (pci), acs ( acute coronary syndrome ), cardiac chest pain, supersaturated oxygen therapy, st elevated myocardial infarction (stemi)

## Abstract

Supersaturated oxygen (SSO_2_) is one of the emerging therapies that has shown benefit for patients suffering from acute ST elevation myocardial infarction (STEMI) in terms of reducing infarct size, which has been used as a prognostic indicator for future heart failure and hospitalizations. Trials investigating SSO_2_ therapy have shown improvement in infarct size when used as an adjunct therapy to percutaneous trans-luminal coronary angioplasty (PTCA) or percutaneous coronary intervention (PCI) in patients presenting with acute myocardial infarction (aMI).

Here we present a patient with a mid left anterior descending artery (mLAD) STEMI who underwent SSO_2_ therapy. The patient presented with new onset angina and ST elevations on EKG. He underwent emergent coronary angiography, which confirmed an mLAD complete vessel occlusion. Successful PCI was done with a drug-eluting stent followed by supersaturated oxygen therapy. On follow-up evaluation, the patient had improved left ventricular (LV) ejection fraction from 35% to 60%.

This case highlights the safety and efficacy of SSO_2_ therapy for patients suffering from acute anterior wall myocardial infarction. We recommend further investigation of this therapy for its routine use, safety, and prognostic utility. We also recommend routine use of adjunctive SSO_2_ therapy for patients suffering acute anterior STEMI.

## Introduction

Acute ST elevation myocardial infarction (STEMI) is a major contributor to overall cardiovascular morbidity and mortality. In 2021, the CDC data showed 5% of adults in the United States were diagnosed with coronary heart disease. Heart disease was ranked as the number one cause of death and had 695,547 deaths attributed to it. However, early reperfusion using primary coronary intervention (PCI) has led to a decline in cardiovascular mortality [1]. When PCI was compared to thrombolytic therapy, infarct size, reinfarction, stroke, and death showed superior outcomes [2,3,4]. Despite these advancements, patient recovery, overall cardiac functionality, and long-term mortality can remain affected following primary intervention through left ventricular remodeling, especially for those with preexisting diminished left ventricular performance [5,6]. Infarct size has been found to have a strong association with mortality and hospitalization following PCI to the extent that it is often used as a prognostic indicator [7]. This is due to mitochondrial dysfunction from reperfusion and microvascular obstructions of the infarcted area resulting in myocardial remodeling [8,9]. A new therapy, supersaturated oxygen therapy (SSO_2_), that acts by potentially improving microvascular flow after the epicardial flow has been restored with PCI has shown some promise. It delivers hyperoxemic levels of oxygen (7-10 times normal) delivered to the myocardium to salvage ischemic tissue. Here we describe one of the first few cases in the state of West Virginia that received this therapy and had a good outcome. The patient presented with acute anterior STEMI with occluded left anterior descending (LAD) artery and reduced ejection fraction who received adjunctive SSO_2_ therapy after epicardial flow was restored with coronary angioplasty and stent placement.

## Case presentation

**Figure 1:**
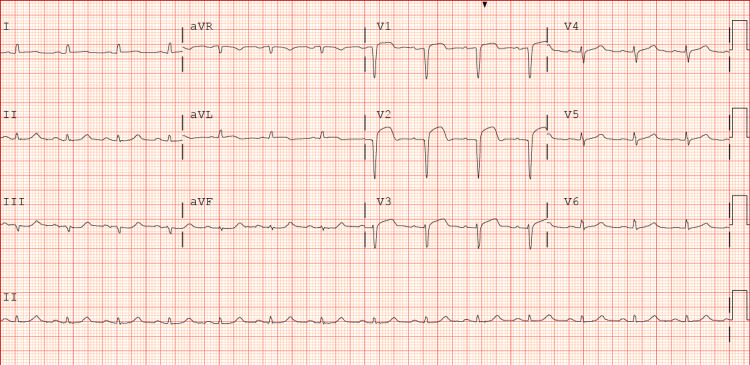
EKG with ST elevation in anterior precordial leads

A 58-year-old male patient with a past medical history of hypertension and diabetes presented to the hospital with chest pain. Chest pain started at 9am while he was at work, and he came to the ER around 11:30am. He characterized his chest pain as dull, 7/10 in intensity, and intermittent with radiation to his left shoulder. He denied any association with nausea, vomiting, or diaphoresis. An EKG (Figure 1) was done, which revealed changes concerning of anterior ST elevation myocardial infarction (aSTEMI). He was tachycardic with a heart rate of 116 and hypertensive with a blood pressure of 165/98 on presentation, which remained high till after stent placement.

**Figure 2:**
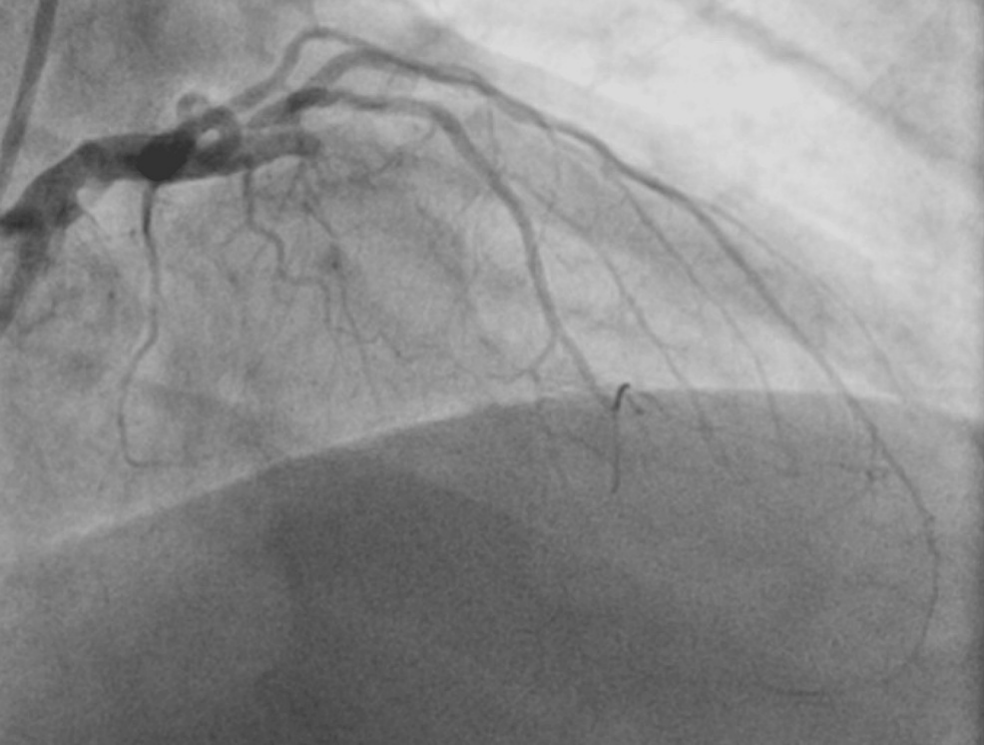
Initial picture depicting complete occlusion of left anterior descending (LAD) artery

The patient was emergently taken to the cardiac catheterization lab for heart catheterization. Angiographic data revealed 100% occlusion of the mid left anterior descending artery (mLAD) (Figure 2), and we did a percutaneous transluminal coronary angioplasty (PTCA) of the culprit lesion at around 12:40pm. 

**Figure 3:**
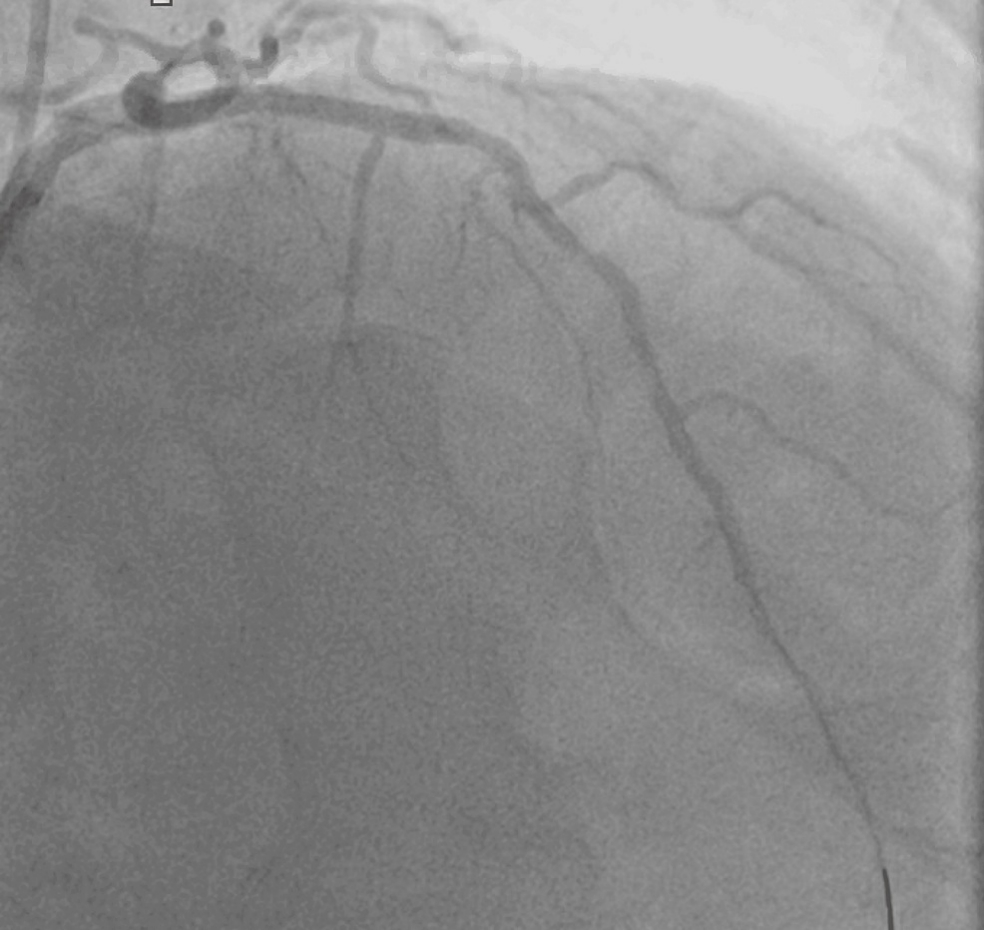
Final picture of left coronary angiogram showing patency of mid left anterior descending (LAD) after balloon angioplasty and stent placement

It was treated with balloon angioplasty and coronary intervention with drug eluting stent placement which helped restore the epicardial flow (Figure 3). His chest pain, which was moderate till now, started to ease off after the patency was established in mLAD. After fixing the LAD, we decided to administer the SSO_2_ therapy to the patient. Right groin access was obtained, and a six French sheath was placed for blood draw. A diagnostic catheter was advanced through the right femoral sheath to engage the Left Main coronary artery for therapy with SSO_2_. Supersaturated oxygen infusion was delivered to the patient for a total of 60 minutes. After treatment, final images were taken, and the patient was transferred to the intensive care unit.

Initial echocardiogram revealed hypokinetic mid anterior septum and apical segment with estimated ejection fraction of about 35% (Video 1). The patient was started on goal directed medical treatment for coronary artery disease (CAD) and left ventricle systolic dysfunction (LVSD) and was discharged home after 48 hours of observation in the hospital. His discharge medications included aspirin, ticagrelor, statin, losartan and metoprolol succinate along with metformin and sitagliptin. 

**Video 1:** Echocardiography showing reduced ejection fraction

On three month follow-up, the patient's repeat echocardiogram revealed that the ejection fraction had improved to about 60% with improved wall motion of the anterior wall and apex.

## Discussion

Despite improvements in morbidity and mortality following early reperfusion with percutaneous coronary intervention (PCI), degradation of myocardium and decline in left ventricular function continues to impact those following acute myocardial infarction (MI). A relationship between supersaturated oxygen and reduction in cardiac complications and hospitalization, as well as improvement in infarct size and left ventricular function, is being established with a few clinical trials like AMIHOT-I, AMIHOT-II, and IC-HOT mentioned below. Supersaturated oxygen (SSO_2_) therapy uses an extracorporeal high-pressure oxygen infusion system to direct hyper oxygenated blood to the coronary arteries. Early studies of hyperoxemic reperfusion were successful in showing improvement in global wall motion, increase in left ventricular ejection fraction, prevention of LV remodeling, and quicker microvascular reperfusion for those with anterior STEMI [10-12]. The body of research investigating outcomes following adjunctive supersaturated oxygen therapy has shown reduced infarct size and reduction in adverse cardiac outcomes when compared to their controls who received standard therapy without supersaturated oxygen infusion [13,14]. The AMIHOT-I trial originally concluded that hyperoxemic reperfusion did not reduce infarct size except in a subgroup of patients that were treated within six hours of symptom onset [13]. The findings from this trial led to the AMIHOT-II trial, which showed that intracoronary delivery of SSO_2_ can reduce infarct size in some patients treated early for large STEMI without inferior outcomes for major adverse cardiovascular events (MACE). SSO_2_ delivery lasted 90 minutes using the extracorporeal circuit. The follow-up time monitored for infarct size was 14 days, and major adverse cardiovascular events were monitored at 30 days. Patients who received SSO_2_ therapy had greater improvement in regional wall motion, greater resolution of ST segment, and smaller infarct size as measured by technetium-99m-sestamibi single photon emission CT (SPECT). The infarct size among controls was 25%, whereas patients treated with SSO_2_ therapy had an infarct size of 18.5%. Despite the reduction in MACE, the AMIHOT-II trial did produce higher safety events related to hematoma formation than controls [14].

Following the AMIHOT-II trial, studies regarding the safety of the procedure were conducted and showed that delivery of SSO_2_ over 60 minutes through a five French catheter following PCI could successfully reduce infarct size where the previous trials had infusion for 90 minutes with a larger, seven French catheter [15]. The IC-HOT study evaluated the use of SSO_2_ therapy from a safety perspective with guidance from the Food and Drug Administration (FDA) for approval of the therapy. It demonstrated improved clinical outcomes with a reduction in net adverse clinical events (NACE) at 30 days. The study included 100 patients with occlusion of the mid-LAD who received a 60-minute infusion with five French catheter following PCI. Treatment with SSO_2_ therapy met the primary safety endpoint of reduction in NACE of 7.1%, which was below the goal of 10.7%, while producing a reduction in infarct size measured by cardiac magnetic resonance at 30 days: 24.1% in controls and 19.4% in SSO_2_ treated patients. Further investigation of the IC-HOT study patient population did not show differences between groups in one-year follow-up rates of all-cause death, new-onset heart failure (HF), or hospitalization for HF when compared to similar patients in the INFUSE-AMI study. Cardiovascular mortality was 4% in controls, and 0% in treated patients (p=0.04), new onset HF or HF hospitalizations were 7.4 in controls and 0% in treated patients (p=0.01), there was no difference in re-infarction or target vessel revascularization between the two groups, and stent thrombosis was 4.9% in controls and 1.2% in treated patients (p=0.17) [16,17].

SSO_2_ therapy is currently the only approved adjunctive treatment for PCI to produce meaningful reductions in infarct size [18]. However, other approaches have been investigated for the reduction of infarct size, including the use of Impella devices, exposure of the patient to systemic hypothermia, the use of intranasal supplemental oxygen, and the use of pharmacologic agents such as adenosine. Supplemental oxygen delivered through a nasal cannula (NC) has been examined for the potential to decrease infarct size in the AVOID trial and failed to document a difference with the supplemental oxygen compared to controls. The difference in efficacy of reducing infarct size has been attributed to SSO_2_'s ability to titrate oxygen to a higher saturation than supplemental NC oxygen therapy [16]. The use of Impella for LV unloading with early or delayed reperfusion has shown some possible benefit, though appropriately powered data is still lacking to support its use versus standard of care, and the technique attempted has delayed time between presentation and PCI [19]. Systemic hypothermia of 33 degrees Celcius initiated prior to revascularization has yet to have an adequately powered study to fully evaluate therapeutic potential. Even if there was significant use potential, this technique also has significant drawbacks, including patient discomfort, the requirements for additional anti-shivering medication, and hypothermic effects on the coagulation cascade, potentially resulting in platelet activation. High-dose adenosine delivered intracoronary during PCI has also failed to show a reduction in infarct size or microvascular obstruction, as documented in the REFLO-STEMI trial. Additionally, adenosine led to higher rates of adverse clinical events [20,21].

Major limitations of existing research include small sample sizes within the AMIHOT trials and IC-HOT study. The IC-HOT study was open-label and used as a study for device safety and was not powered to test the ability to reduce infarct size when compared to controls; it did use different duration of infusion, 60 minutes as opposed to 90, and smaller catheters, five French as opposed to seven French [13-15]. A follow-up study showed a one-year improvement in clinical outcomes; however, the data was derived from the original population of patients used in the IC-HOT study without an increase in sample size [17].

Our case supports the benefit and safety of SSO_2_ therapy in patients with anterior STEMI as adjunctive treatment with the potential to improve patient outcomes following PCI. Patients with anterior myocardial infarction should continue to receive this treatment in the acute setting to decrease overall major adverse cardiac events and hospitalization. Clinical trials with larger sample sizes that are adequately powered and randomized are warranted to further investigate SSO_2_'s potential to reduce infarct size and, therefore, major adverse cardiac events and hospitalization following PCI for anterior STEMI. Future studies should also investigate longer follow-up times for the determination of greater benefits and limitations.

## Conclusions

This case highlights the safety and efficacy of SSO_2_ therapy for patients suffering from anterior wall MI. There were no procedural complications during or after TherOx SSO_2_ treatment. The patient had an excellent outcome of improved left ventricular ejection fraction at the three-month follow-up. Based on our experience, we would recommend routine use of SSO_2_ treatment for patients with anterior STEMI in addition to guideline directed medical therapy.
